# Differences and Compatibility between Human and Porcine Fibrinolytic Components toward Plasmin Generation and Fibrin Degradation

**DOI:** 10.1055/a-2777-7484

**Published:** 2026-01-09

**Authors:** Paul Y. Kim, Chengliang Wu, Hena Noorzada, Ali Aftabjahani

**Affiliations:** 1 Thrombosis and Atherosclerosis Research Institute, Hamilton, Canada; 2 Department of Medicine, McMaster University, Hamilton, Canada; 3 Department of Medical Sciences, McMaster University, Hamilton, Canada

**Keywords:** swine, fibrinolysis, plasminogen, plasminogen activators, proteolysis

## Abstract

**Abstract:**

**Background:**

Fibrinolysis is the process of blood clot breakdown by the enzyme plasmin. Despite increased usage of large animals such as pigs to study fibrinolysis in human disease models, a comprehensive study comparing the human and porcine fibrinolytic factors has not been reported.

**Objective:**

To directly compare and characterize structural and functional differences between human and porcine fibrinolytic factors.

**Methods:**

Using human or porcine source of plasminogen, tissue-type plasminogen activator (tPA), and fibrinogen, we investigated how various permutations of the three fibrinolytic factors affect overall plasmin generation. Human or porcine plasmin breakdown of fibrin generated from human or porcine fibrinogen was also investigated using turbidity-based lysis assay and visualized using SDS-PAGE. Primary structures of the various proteins were also compared.

**Results:**

All-human components had a 24-fold higher plasmin generation than all-porcine components. Species dependence on plasmin generation was the most dependent on fibrin source, where human fibrin presence led to a 2- to 34-fold higher plasmin generation than porcine fibrin. Porcine plasmin was the better enzyme for human or porcine fibrin breakdown due to a 2.7-fold and 6.7-fold higher k _cat_ , respectively. Peptide sequence analyses show the greatest differences lie in Kringle domain 1 for plasminogen and Kringle domain 2 for tPA, both of which bind fibrin. Fibrinogen chains also show the greatest difference within the αC domain, which has known plasminogen and tPA binding sites.

**Conclusion:**

Although similar, there are notable and specific differences between the human and porcine fibrinolytic systems, particularly toward plasmin generation and fibrin breakdown.

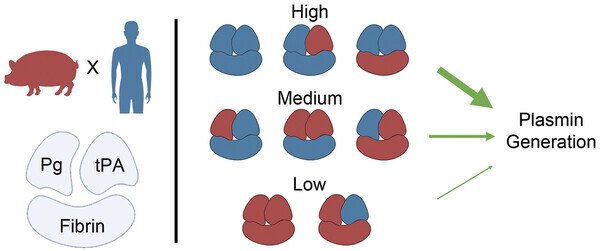

## Introduction

Fibrinolysis, the biological process of enzymatically breaking down insoluble fibrin clots to soluble fibrin degradation products (FDPs) is initiated by the activation of the zymogen plasminogen into the central enzyme plasmin. This reaction is mediated by the action of upstream enzymes tissue-type plasminogen activator (tPA) or urokinase-type plasminogen activator (uPA), depending on where the reaction is taking place. 1 2 A key characteristic that distinguishes tPA from uPA is that tPA demonstrates affinity for fibrin while uPA does not. 3 The binding of tPA to fibrin through its finger domain and Kringle 2 domain allows for the formation of the tPA/plasminogen/fibrin complex for efficient activation of plasminogen to plasmin. 4 5 6 The presence of fibrin enhances the overall catalytic efficiency of plasmin generation by three orders of magnitude compared with no fibrin, thus making fibrin an important cofactor for its own breakdown. 7 Fibrinogen, the soluble monomeric precursor of fibrin, can also promote plasmin generation at a rate that is 10-fold lower than with fibrin. 8 This specificity allows for localized plasmin generation on fibrin clots, promoting effective clot dissolution while limiting systemic degradation of fibrinogen. In contrast, the catalytic efficiency of plasmin generation by uPA is comparable to that of tPA in the presence of fibrinogen as a cofactor. 8 9

The predominant and full-length form of plasminogen that is found in circulation is often referred to as Glu-Pg, which refers to its N-terminal amino acid being Glu1. Plasminogen is comprised of seven major domains: N-terminal peptide, five Kringle domains, and the serine protease domain. 10 Of these, Kringle domains 1, 4, and 5 possess fibrin-binding properties in a lysine-dependent manner (i.e., binds to either internal or terminal lysine residues in proteins). 11 12 13 14 Activation of plasminogen involves the proteolytic cleavage at Arg561 within the protease domain of plasminogen, thus generating a heavy chain and a light chain that are held together by a disulfide bridge. 10 15

Breakdown of human fibrin clots by human plasmin has been studied extensively. 16 17 18 19 20 Single molecule of fibrinogen is composed of six independent polypeptides—two of alpha, beta, and gamma chains—that are held together by numerous disulfide bridges. 16 21 The two halves of the molecule mirror each other with the N-termini all located at the center of the molecule and the C-termini extending toward opposing distal ends. Once fibrinogen is cleaved by thrombin, each fibrin monomer can aggregate with other fibrin monomers in a half-staggered, brick-laying orientation to generate a double-layered protofibril. 22 Thus, in order to cleave through a single protofibril strand, at least six polypeptides must be cleaved at a specific location to achieve full dissolution. Plasmin degrades fibrin via specific sites to generate consistent fibrin degradation products (FDPs), where the smallest fragment generated is the DD(E) fragment, containing two of the distal C-terminal ends (D domains) that are cross-linked (D-dimer) with a non-covalently associated central N-termini region (E domain) of the opposing strand. 16 23 Breakdown of individual chains of fibrin demonstrated similar rates of cleavage, suggesting a coordinated and localized cleavage of each protofibril rather than randomized cleavage at various locations. 16 24

The fundamental mechanisms of fibrinolysis are conserved across species. As such, fibrinolysis has been and continues to be investigated with various animal models. Historically, small rodents have been used the most, and as a consequence, notable differences have been reported. 25 26 Underestimation of the subtle differences may result in different outcomes when similar experiments are performed across different species. This has been shown to be true, even when mixing clotting components from different species to study thrombin generation. 27 Particularly, understanding incompatibility when mixing fibrinolytic components from different species is critical for the success of experiments. Recently, increasing number of studies have been using large animals to investigate how the human circulatory systems and associated organs may respond to various insults. Particularly pigs have been used due to their similarities with humans in terms of size and makeup of its circulatory system. 28 29 30 31 32 33 Our study characterizes the specific contribution and compatibility between porcine or human fibrinogen, plasminogen, and tPA in overall plasmin generation. These findings are crucial to better interpret the results obtained from porcine model–based preclinical studies and better anticipate how they may be translated to human subjects.

## Experimental Procedures

*Materials:* Recombinant porcine tPA, recombinant porcine uPA, purified porcine fibrinogen, and purified porcine plasminogen were purchased from Innovative Research Inc (Novi, MI, USA). Human tPA was purchased from Kingston General Hospital Pharmacy (Kingston, ON, Canada). Batroxobin was purchased from DSM Nutritional Products Ltd. Branch Pentapharm (Aesch, BL, Switzerland). Unless otherwise stated, experiments were performed using 0.02 M HEPES, 0.15 M NaCl, pH 7.4 (HBS) with 0.01% Tween-80 (HBST). All microtiter plates that were used were clear flat-bottom polystyrene 96 well plates (Greiner Bio-One) that were pre-treated for at least 5 hours with HBS containing 1% Tween-80 and washed thoroughly with water and air-dried prior to use. SpectraMax M3 plate reader (Molecular Devices, Sunnyvale, CA, USA) was used to measure turbidity and absorbance. Glu-Pg was prepared in-house for experimental use as described previously. 8 Lysine analog ε-aminocaproic acid (εACA) was obtained from Millipore Sigma. Chromogenix S-2251, a substrate for plasmin activity assays, was obtained from DiaPharma. For SDS-PAGE analysis, Mini-PROTEAN TGX Gels were purchased from Bio-Rad. Human alpha-thrombin and fibrinogen were purchased from Enzyme Research Laboratories (South Bend, IN, USA). Prior to use, residual levels of factor XIII were removed from fibrinogen using affinity chromatography and intact fibrinogen was isolated using ammonium sulfate precipitation as described previously. 16 34 Porcine fibrinogen was dialyzed against HBST prior to use. Lack of residual factor XIIIa activity was confirmed by thrombin-mediated clotting and visualization via SDS-PAGE.

*Clotting porcine fibrinogen using human thrombin:* Using a 96-well microtiter plate, porcine fibrinogen at varying concentrations (0 to 7.5 μM) was clotted with human thrombin (1 nM) in the presence of CaCl _2_ (5 mM) in HBST. Briefly, 5 µL of thrombin was placed in the corner of a 96-well microtiter plate, and the reaction was initiated by addition of a 95 µL mixture containing porcine fibrinogen and CaCl _2_ at 37°C. Turbidity was monitored at 1 minute interval using the M3 SpectraMax plate reader at 400 nm. Clot time was determined as the time required to reach half-maximal turbidity change from when the reaction was initiated as described previously. 24 The clot times were then plotted as a function of initial fibrinogen concentration.

*Preparation of porcine plasmin:* Porcine plasmin was generated in-house to determine its specific activity toward the chromogenic substrate S-2251. Briefly, porcine plasminogen (750 nM) was incubated with porcine uPA (100 nM) in the presence of εACA (10 mM) in HBST, similar to the protocol as described in generating human plasmin. 35 The reaction mixture was incubated at 25°C for 30 minutes. The product was then dialyzed at 4°C against HBST to remove εACA. The resulting plasmin solution was then visualized using SDS-PAGE and its concentration was determined by quantitative densitometry. 36

Using the estimated plasmin concentration, its specific activity toward S-2251 was assessed as described previously. 8 37 Briefly, plasmin was added at varying concentrations (0, 5, and 10 nM) into S-2251 (500 µM) in HBST. Cleavage of S-2251 was monitored at 405 nm using the SpectraMax M3 plate reader at 25°C, with the readout set for 2 hours at 1-minute intervals. The initial rate was determined using SoftMax Pro (v7) and plotted as a function of plasmin concentration. The slope represents the specific activity.

*Comparison of kinetics of plasminogen activation between porcine and human systems:* To determine compatibility of plasminogen, fibrinogen, and tPA between porcine and human systems, plasmin generation from permutation of all possible combinations was investigated using a plasmin-specific chromogenic substrate as described previously, 8 38 with slight modifications. Briefly, human thrombin (2 nM) and human or porcine tPA (0.1 nM) were placed in the opposing corners of each well in a 96-well microtiter plate. Clot formation and lysis reactions were initiated by the addition of a mixture solution containing human or porcine plasminogen (0, 0.1, 0.25, 0.5, or 0.75 µM), fibrinogen (1 µM), S-2251 (250 µM), and CaCl _2_ (5 mM) in HBST. Plasmin generation was determined using the M3 SpectraMax plate reader by monitoring at 405 and 600 nm at 37°C in 15 s intervals for 2 hours. The resulting turbidity profiles were then normalized to only show S-2251 hydrolysis by plasmin and rates were calculated using time-squared analysis. The rates calculated were then plotted with respect to initial plasminogen concentration. The K _m_ and k _cat_ values of each reaction condition were estimated. Plots approaching saturation were evaluated by fitting the data to the rectangular hyperbola equation (i.e., Michaelis-Menten equation) using SigmaPlot (v11.0) and the catalytic efficiency was calculated by dividing the k _cat_ by K _m_ . With linear plots, the slope was estimated as catalytic efficiency.

*Evaluation of fibrin clot breakdown by plasmin:* To determine if human or porcine plasmin demonstrates different proteolytic characteristics toward human or porcine fibrin, clots were generated and their breakdown patterns analyzed using SDS-PAGE as described previously. 24 Since fibrinogen has α _2_ -antiplasmin (α _2_ AP) that is cross-linked to it, the level of plasmin inhibition activity toward either human or porcine plasmin was estimated as described previously, 24 by titrating increasing concentrations of human or porcine fibrinogen into 10 nM human or porcine plasmin.

Once the α _2_ AP activity content was determined, clot degradation patterns were analyzed by SDS-PAGE to see if degradation patterns were different based on the species of fibrinogen and/or plasmin. Briefly, 5 µL of batroxobin (2 U/mL final) and 5 µL of human or porcine plasmin (5 nM active final) were placed in the opposing corners of each well in a 96-well microtiter plate. The reactions were initiated by the addition of a 65 µL mixture containing human or porcine fibrinogen (3 µM) with CaCl _2_ (5 mM) in HBST. For each reaction condition, at least six identical clots were formed to generate a time course of the reaction. The clotting and lysis reactions were monitored spectrophotometrically at 400 nm. Reactions were stopped by the addition of 35 µL of 0.3 N acetic acid into individual wells and mixed vigorously at three time points: right after clot has fully formed, when the OD reached half-maximal decrease during lysis, and at complete lysis. 24 The protein degradation profiles were then resolved using SDS-PAGE and visualized with Coomassie blue.

To determine the kinetics of plasmin-mediated fibrin degradation from different species combination of plasmin and fibrin, clot lysis times were measured as described previously. Because even a small amount of factor XIIIa activity of cross-linking fibrin could impact the kinetics of fibrin breakdown, batroxobin was used to generate the fibrin clots instead of thrombin. Briefly, 5 µL of batroxobin (2 U/mL final) and 10 µL of human or porcine plasmin (5 nM active final) were placed in the opposing corners of each well in a 96-well microtiter plate. The reactions were initiated by the addition of a 85-µL mixture containing human or porcine fibrinogen at varying concentrations (0 to 5 µM) with CaCl _2_ (5 mM) in HBST. Clot lysis time was determined as the time required to reach half-maximal change in OD during degradation from when the reaction was started. 24 39 To determine the k _cat_ and K _m_ of global fibrin breakdown by plasmin, the slope of lysis time as a function of fibrinogen concentration was fit to the equation  , as described previously, where [Fn] _0_ is the starting fibrinogen concentration, [Pn] is the active total concentration of plasmin, k _cat_ is the turnover rate constant, and K _m_ is the Michaelis constant. 24

*Comparison of primary sequence structures of fibrinogen, plasminogen, and tPA between and human and porcine factors:* Pairwise alignment was performed using EMBOSS Needle (v6.6.0) (EMBL's European Bioinformatics Institute) with the BLOSUM62 substitution matrix, a gap open penalty of 10, and a gap extension penalty of 0.5, no end gap penalty, an end gap open penalty of 10, and an end gap extend penalty of 0.5. Plasminogen (human—UniProtKB:P00747; porcine—UniProtKB:P06867), tPA (human—UniProtKB:P00750; porcine—UniProtKB:Q8SQ23), or fibrinogen (alpha chain [human—NP_000499.1; porcine—XP_020957142.1]; beta chain [human—NP_005132.2; porcine—NP_001231042.1]; gamma chain [human—NP_000500.2; porcine—NP_001231453.1]) sequences were compared. Alignment was visualized using the Multiple Align Show Tool to evaluate conservation across functional domains. Similarities and identities of each protein, including the signaling peptide, as well as comparisons by individual functional domains were performed. Annotation of domain boundaries were based on established databases and literature.

*Statistical analysis:* Statistical analyses were conducted using RStudio (2025.05.1 Build 513). Data are presented as the mean ± standard error. Two-way and three-way analysis of variance (ANOVA) was used to compare multiple groups with multiple conditions. When statistical significance was identified (defined as *P* < 0.05) in the factorial ANOVAs, interaction and simple effects analyses were performed to determine interspecies interactions. When statistical significance was not found in the factorial ANOVAs, simple main effects analyses were performed.

## Results

**Fig. 1:**
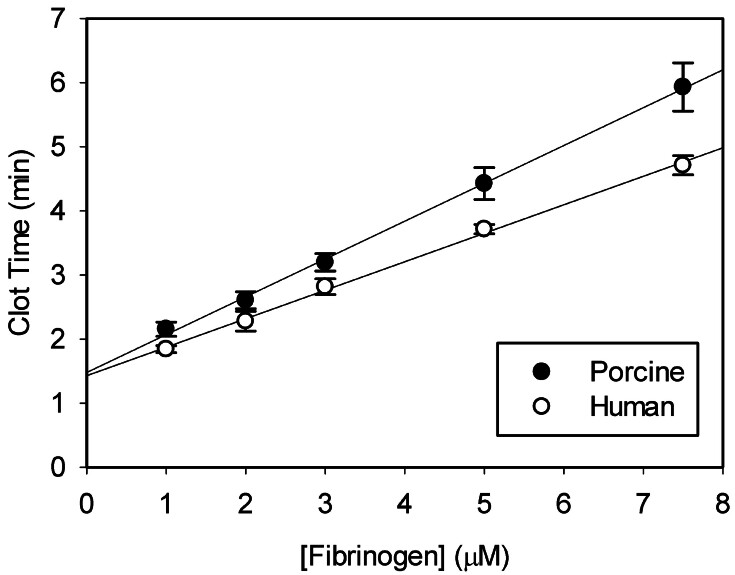
Clotting of purified fibrinogen by human thrombin. Porcine ( *closed* ) or human ( *open* ) fibrinogen at varying concentrations (1 to 7.5 µM) was clotted with human thrombin (1 nM), and the reaction was observed by turbidity. Clot times were determined as the time required to reach half-maximal increase in turbidity from the start of the reaction. The error bars represent the mean ± SEM ( *N* = 3).

*Porcine fibrinogen is sensitive to human thrombin for clotting:* To determine if porcine fibrinogen is sensitive to clotting by human thrombin, clot times of porcine fibrinogen at varying levels was determined using human thrombin ( Fig. 1 ). Clot times increased with increasing concentrations of initial fibrinogen, suggesting that porcine fibrinogen is sensitive to human thrombin in a concentration-dependent manner, similar to human fibrinogen.

**Fig. 2:**
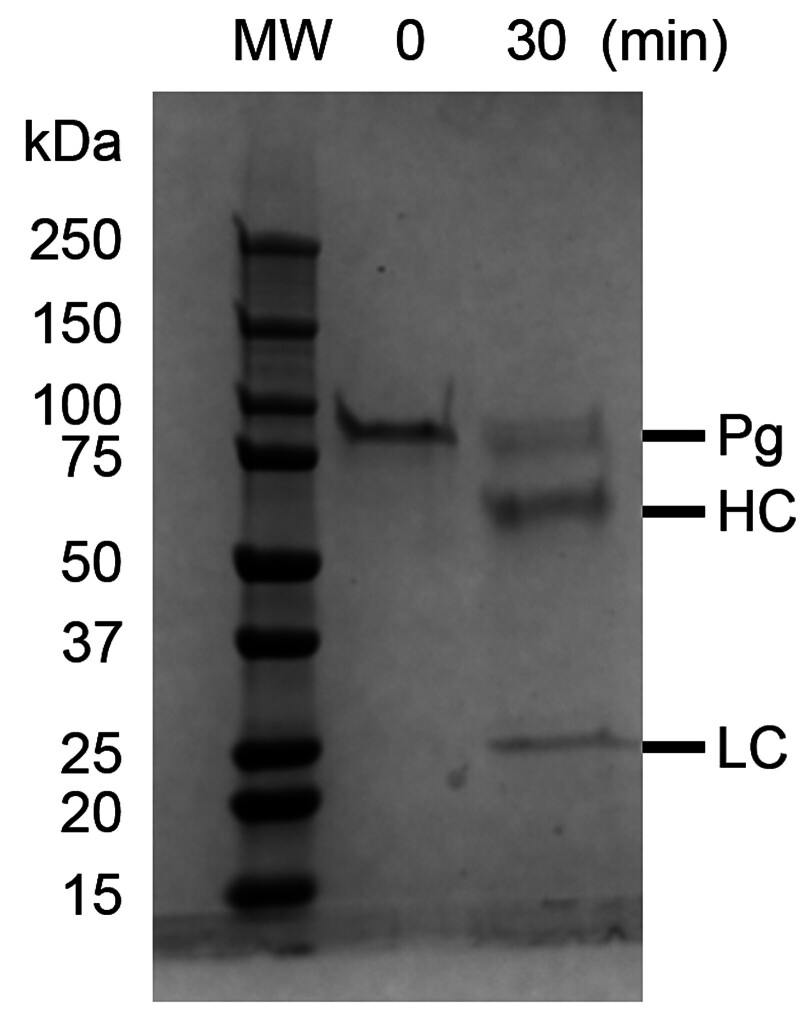
SDS-PAGE analysis of porcine plasminogen activation by porcine uPA. Porcine plasminogen (750 nM) was incubated with porcine uPA (100 nM) in the presence of εACA (10 mM) for 0 min ( *left lane* ) or 30 min ( *right lane* ) at 25°C. Samples were taken to resolve the bands on SDS-PAGE (5–15% gradient) and quantitative densitometry was performed to estimate the proportion of plasminogen that was converted to plasmin. HC, heavy chain; LC, light chain; Pg, plasminogen.

*Comparison of kinetics of plasmin generation by tPA between porcine and human fibrinolytic systems:* To quantify the kinetics of plasmin generation, the respective specific activities of human or porcine plasmin toward the chromogenic substrate S-2251 were experimentally determined. To do so, we first generated and isolated porcine plasmin by activating porcine plasminogen with porcine uPA. To determine the concentration of plasmin, SDS-PAGE and quantitative densitometry was performed ( Fig. 2 ), where the activation protocol used resulted in approximately 72% conversion of porcine plasminogen to plasmin. Using the established plasmin concentration, the specific activity of porcine plasmin toward S-2251 was determined to be 1.05 ± 0.01 A _corr_ /min/µM. The specific activity of human plasmin was determined to be 1.21 ± 0.02 A _corr_ /min/µM, comparable to that reported previously. 8 38

**Fig. 3:**
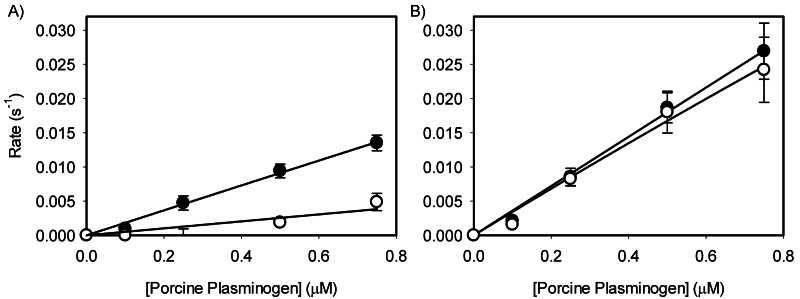
Rates of porcine plasmin generation by porcine (closed) or human (open) tPA. Porcine plasminogen (Pg) at varying concentration (0 to 0.75 µM) was activated by tPA (0.1 nM) in the presence of either (A) porcine or (B) human fibrin (1 µM); converted from fibrinogen using human thrombin (2 nM). The initial rates of plasmin generation were then estimated using the time-squared method 8 and plotted with respect to the initial plasminogen concentration ( *N* ≥ 3).

Using the experimentally determined specific activity, initial rates of porcine or human plasmin generation was quantified, and their overall catalytic efficiencies were estimated as described previously. 8 First, we compared porcine plasmin generation ( Fig. 3 ). With porcine fibrin as the cofactor, the catalytic efficiency of porcine plasminogen activation by porcine tPA was 5.5-fold faster than with human tPA ( *P* = 0.0001) ( Fig. 3A , Table 1 ). With human fibrin ( Fig. 3B ), activation of porcine plasminogen was not different ( *P* > 0.7) between porcine and human tPA, eliminating the species dependence of tPA seen with porcine fibrin. In addition, porcine plasmin generation by human tPA on human fibrin was 10-fold faster ( *P* = 0.008) compared with porcine fibrin.

**Table 1:** Catalytic efficiency of porcine plasminogen activation with varying combination of porcine or human fibrin and tPA (The values represent mean ± standard error; *N* ≥ 3)

tPA	Catalytic efficiency (µM ^−1^ s ^−1^ )		
	Porcine fibrin	Human fibrin	*P*
Porcine	0.018 ± 0.001	0.036 ± 0.005	0.006
Human	0.0033 ± 0.0009	0.033 ± 0.006	0.008
*P*	0.0001	>0.7	

**Fig. 4:**
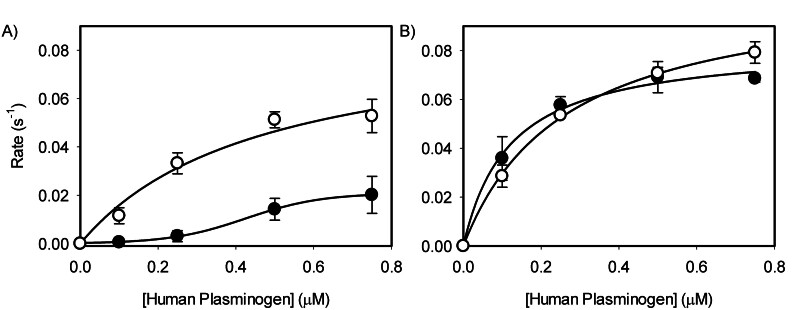
Rates of human plasmin generation by porcine (closed) or human (open) tPA. Human plasminogen (Pg) at varying concentration (0 to 0.75 µM) was activated by tPA (0.1 nM) in the presence of either (A) porcine or (B) human fibrin (1 µM); converted from fibrinogen using human thrombin (2 nM). The initial rates of plasmin generation were then estimated using the time-squared method 8 and plotted with respect to the initial plasminogen concentration ( *N* ≥ 3).

Catalytic efficiency of human plasmin generation by all human system was approximately 24.3-fold greater than porcine plasmin generation by all porcine system ( Tables 1 and 2 ). Activation of human plasminogen was more efficient compared with porcine plasminogen in all combinations ( Tables 1 and 2 ), as observed by the saturating plasmin generation rate curves ( Fig. 4 ). In the presence of porcine fibrin, activation of human plasminogen by human tPA was 7.2-fold faster than with porcine tPA ( *P* = 0.007). The presence of human fibrin resulted in a similar plasmin generation rates between human and porcine tPA ( *P* > 0.24), also eliminating the species dependence of tPA seen with porcine fibrin. The catalytic efficiency of human plasminogen activation by human tPA on human fibrin was approximately 2.3-fold greater compared with porcine fibrin ( *P* = 0.04). Together, our data demonstrate that there are observable differences between the porcine and human fibrinolytic system components, and that human tPA is a poor activator of pig plasminogen on pig fibrin. A three-way ANOVA revealed a significant three-way interaction between the species of tPA, plasminogen, and fibrinogen ( *P* = 0.026). Subsequent two-way interactions revealed that the interaction between species of tPA and fibrinogen was not statistically significant, regardless of the plasminogen species used ( *P* = 0.096 for human plasminogen and *P* = 0.18 for porcine plasminogen). A significant two-way interaction was observed between the species of fibrinogen and plasminogen. In the presence of human tPA, a significant interaction between plasminogen and fibrinogen was observed across all tested combinations ( *P* = 0.026). In the presence of porcine tPA, a significant interaction between plasminogen and fibrinogen was also detected ( *P* = 0.0009). However, a simple effects analysis showed that when porcine fibrinogen was the cofactor, there was no significant effect from species of plasminogen on plasmin generation ( *P* = 0.063). Lastly, plasminogen and tPA interactions revealed that the significance was dependent on the fibrinogen species. A significant interaction between plasminogen and tPA species was found in the presence of porcine fibrinogen ( *P* < 0.0001), as seen in Figs. 3A and 4A (i.e., porcine fibrin[ogen] accentuates species dependence). However, this interaction was not statistically significant in the presence of human fibrinogen ( *P* = 0.11), as seen in Figs. 3B and 4B . Taken together, our data demonstrate the delicate nature of species-specificity of these factors that otherwise show high similarity based at least on their overall primary structures.

**Table 2:** Catalytic efficiency of human plasminogen activation with varying combination of porcine or human fibrin and tPA (The values represent mean ± standard error; *N* ≥ 3)

tPA	Catalytic efficiency (µM ^−1^ s ^−1^ )		
	Porcine fibrin	Human fibrin	*P*
Porcine	0.026 ± 0.010	0.88 ± 0.31	0.0496
Human	0.19 ± 0.03	0.44 ± 0.08	0.04
*P*	0.007	0.24	

*Differences in fibrin breakdown by porcine and human plasmin:* To ensure that equal active concentration of plasmin was present in each of the reaction conditions, levels of α _2_ AP inhibition activity was first experimentally determined. Porcine fibrinogen (1 µM) contained 3.4 and 3.5 nM α _2_ AP activity toward porcine and human plasmin, respectively. Human fibrinogen (1 µM) contained 1.8 and 0.9 nM α _2_ AP activity toward porcine and human plasmin, respectively, consistent with our previous finding. 24

**Fig. 5:**
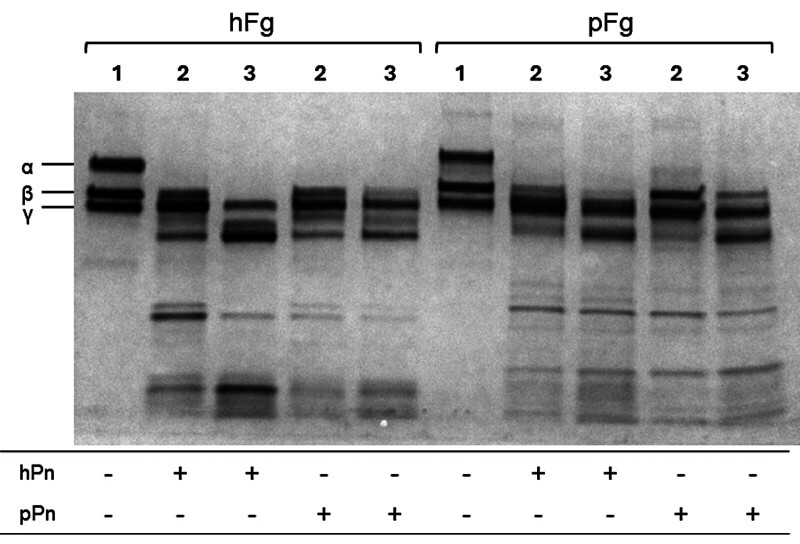
Analysis of fibrin breakdown by plasmin using SDS-PAGE. Identical clots were generated using human fibrinogen (hFg) or porcine fibrinogen (pFg) in 96-well microtiter plates. Clots were formed using batroxobin (2 U/mL) while lysis was initiated by either human (hPn) or porcine (pPn) plasmin (5 nM active). Clot formation and lysis were monitored by turbidity, and the reaction was stopped by the addition of acetic acid (0.3 N) when lysis achieved 50 or 100% degradation. Label #1 shows fibrinogen (pre-clot/lysis); #2 shows at 50% lysis; #3 shows at 100% lysis.

**Fig. 6:**
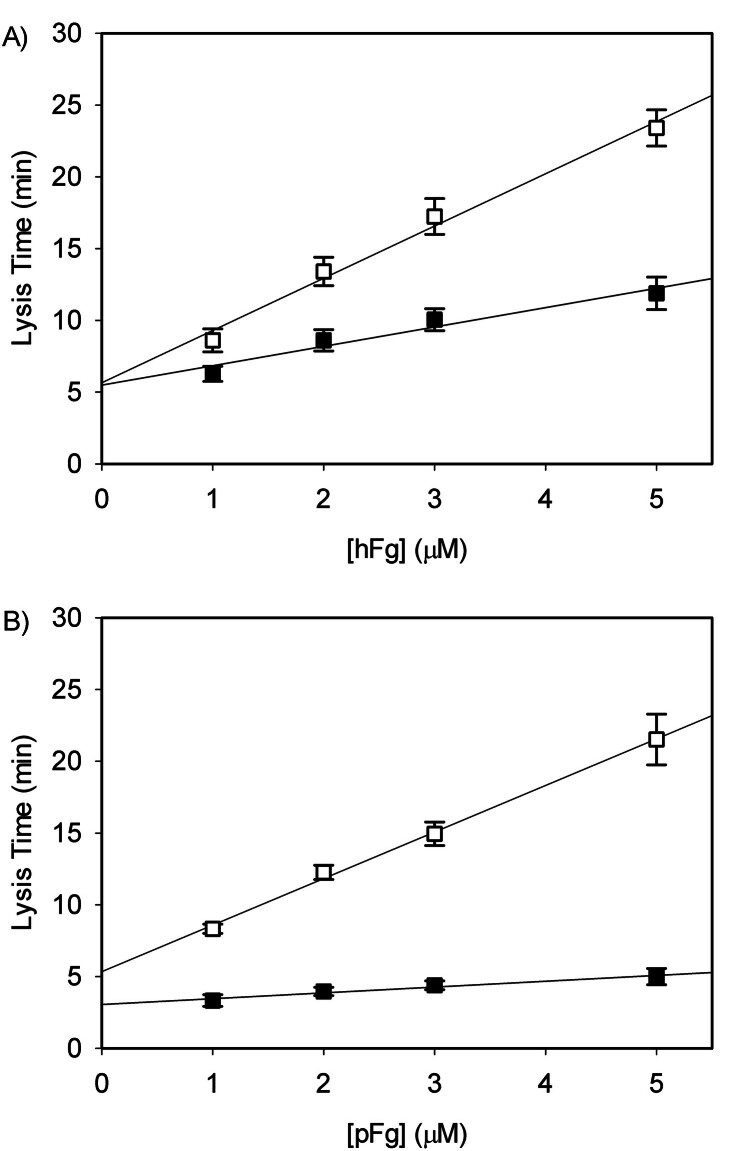
Kinetics of fibrin degradation by porcine ( *closed* ) or human ( *open* ) plasmin. Clots were generated using A) human (hFg) or B) porcine (pFg) fibrinogen at varying concentrations (1 to 5 µM) with the reaction initiated using batroxobin (2 U/mL). Lysis was initiated by plasmin totaling 5 nM final active concentration after correcting for α _2_ AP inhibition from varying fibrinogen levels. Using the equation 24 :  , individual k _cat_ and K _m_ values of fibrin breakdown by plasmin were estimated.

To characterize whether proteolytic cleavage of porcine or human fibrin clot by porcine or human plasmin differed at varying points of clot lysis by turbidity, fragment patterns were compared at 0, 50%, and 100% clot lysis as observed by turbidity using SDS-PAGE ( Fig. 5 ). There were noticeable differences across all combinations. Most notably, human plasmin was able to digest all of the alpha and beta chains in both human and porcine fibrin by 100% clot lysis. Digestion of both fibrins by human plasmin had greater accumulation of the smaller degradation products than porcine plasmin. However, this may simply be due to longer total incubation time of fibrin with human plasmin due to substantially higher lysis time observed when assessed by turbidity compared with porcine plasmin ( Fig. 6 ).

From the regression line estimated from the plot of lysis time as a function of initial fibrinogen concentration ( Fig. 6 ), both k _cat_ and K _m_ values of the overall fibrin breakdown process by plasmin were estimated. 24 For human fibrin clots, human plasmin had k _cat_ and K _m_ values of 0.44 ± 0.02 s ^−1^ and 1.1 ± 0.1 µM ( Table 3 ), respectively, which is consistent with our previous report of 0.47 ± 0.03 s ^−1^ and 1.1 ± 0.6 µM, respectively. 24 Neither human plasmin ( *P* = 0.42) nor porcine plasmin ( *P* = 0.1) demonstrated significant difference toward fibrin species for k _cat_ values based on simple effects. However, fibrin was sensitive to plasmin species whereby porcine plasmin demonstrated greater fibrin breakdown potential than human plasmin toward both human and porcine fibrin ( Fig. 6 ). Analysis of the kinetic parameters ( Table 3 ) revealed no significant interspecies interaction of the K _m_ values ( *P* = 0.37), indicating comparable substrate binding affinity of plasmin to fibrin regardless of species combinations. In contrast, a significant interspecies interaction was observed for k _cat_ ( *P* = 0.016), demonstrating a species-dependent difference in their turnover. Breakdown of this interaction showed that altering the species of fibrin substrate (human versus porcine) did not have statistically significant effect neither on plasmin enzyme efficacy toward human fibrin ( *P* = 0.42) nor porcine fibrin ( *P* = 0.097). However, when analyzing the effect of altering plasmin species, a significant effect was observed on the degradation of human fibrin ( *P* = 0.04) but not porcine fibrin ( *P* = 0.062). When catalytic efficiency was calculated, however, only notable differences were cleavage of porcine or human fibrin by porcine plasmin ( *P* = 0.040) and cleavage of porcine fibrin by either porcine or human plasmin ( *P* = 0.046).

**Table 3:** Kinetics of fibrin breakdown by plasmin

Plasmin	Fibrin	k _cat_ (s ^−1^ )	K _m_ (µM)	C.E. (k _cat_ /K _m_ ) (µM ^−1^ s ^−1^ )
Porcine	Porcine	3.43 ± 0.63	4.9 ± 1.6	0.79 ± 0.13
	Human	1.21 ± 0.13	3.1 ± 0.3	0.40 ± 0.03
Human	Porcine	0.51 ± 0.06	1.3 ± 0.2	0.41 ± 0.04
	Human	0.44 ± 0.02	1.1 ± 0.1	0.44 ± 0.06

Note: The values represent mean ± standard error ( *N* ≥ 3). NS, *P* ≥ 0.05; *, *P* < 0.05.

**Fig. 7:**
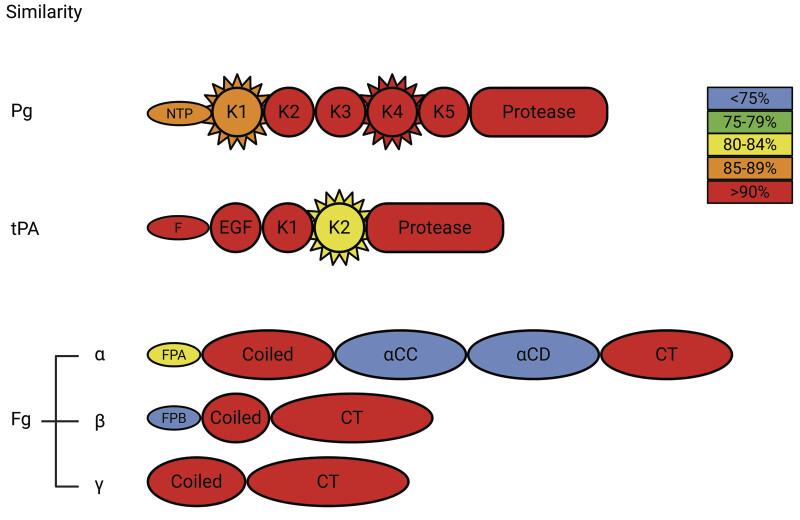
Similarity of amino acid sequences when compared between porcine and human plasminogen, tPA, and fibrinogen chains by domain. The heat map illustrates domains that display highest (red) to lowest (blue) similarities. The spikes highlight the domains that bind fibrinogen in a lysine-dependent manner. (Created in BioRender. Kim, P. (2025) https://BioRender.com/zvi11gx .)

*Comparison of primary structures of plasminogen, tPA, and fibrinogen between human and porcine:* To determine if the differences in plasmin generation and consequent fibrinolysis when mixing porcine and human systems can be attributed to their primary structures, we compared the peptide sequences of plasminogen, tPA, and fibrinogen. Plasminogen ( Supplementary Fig. S1 ) shows 80% identity ( Supplementary Fig. S6 ) and 88% similarity ( Fig. 7 ), with one residue omitted out of 810 total residues. Further analysis of individual domains highlighted that the biggest difference between the two species are localized to Kringle domain 1 with similarity of 84.8% compared with K2, K3, K4, and K5, and the protease domain all having similarity above 90% ( Supplementary Table S1 ). The linker region between K4 and K5 containing the gap residue also demonstrated poor sequence conservation, which is notable since Kringle domains 1 and 4 are thought to be most important for fibrin binding. 11 12 13 14

Comparison of tPA ( Supplementary Fig. S2 ) shows 82% identity ( Supplementary Fig. S6 ) and 89% similarity ( Fig. 7 ), with no gaps in the sequence. Further analysis of individual domains also shows that the biggest difference between the two species lies within the Kringle 2 domain, which is also the fibrin-binding domain 40 41 ( Supplementary Table S2 ). Closer inspection of the primary structure showed mutations of residues KNRR in human to KDHK in porcine, where either of the Arg77 or Arg 78 of the K2 sequence is thought to be important for lysine binding. 42 43

Comparison was also made for fibrinogen chains. Alpha chain ( Supplementary Fig. S3 ) comparison resulted in 65% identity ( Supplementary Fig. S6 ) and 73% similarity ( Fig. 7 ), with 98 gap residues (10.4%) in the sequence. Closer inspection of the alpha chain domains demonstrated that the biggest difference between the two species lies within the αC region (i.e., αC connector and αC domain), 44 45 which is thought to possess a tPA/plasmin(ogen) binding site 44 46 47 ( Supplementary Table S3 ). The αC region also contained 70 of the 98 gap residues, while FPA had 1, and the other 27 due to extended signal peptide N-terminal portion of the porcine sequence. These demonstrate that 98.6% (70/71) of the gaps within functional region of fibrinogen alpha chain are located withing the αC region. All other domains showed sequence similarity that is greater than 93%.

Beta chain ( Supplementary Fig. S4 ) comparison resulted in 82% identity ( Supplementary Fig. S6 ) and 90% similarity ( Fig. 7 ), with 10 gap residues (2%) in the sequence ( Supplementary Table S4 ). Fibrinopeptides A and B shown for porcine sequence are assumed based on alignment with human sequence, despite differences in peptide length, particularly in fibrinopeptide B.

Gamma chain ( Supplementary Fig. S5 ) comparison resulted in 83% identity ( Supplementary Fig. S6 ) and 93% similarity ( Fig. 7 ), with one gap residue (0.2%) in the sequence ( Supplementary Table S5 ). Taken together, the greatest difference in fibrinogen is localized to the αC region within the alpha chain.

## Discussion

Direct comprehensive comparison of the porcine and human fibrinolytic factors and their individual contribution to overall plasmin generation has not yet been reported. This is of particular interest since pigs are being used more frequently to model human systems and their associated diseases due to their similarities in genetic compatibility, as well as other physical attributes such as size and blood volume. In a comparative study using porcine and human blood *in vitro* , researchers investigated the responsiveness to a novel fibrinolytic inhibitor to evaluate the suitability of pigs as animal models for *in vivo* testing. 48 Thromboelastography revealed that clots formed from porcine whole blood exhibited high resistance to tPA catalyzed lysis, attributed to the resistance of porcine plasminogen to tPA-mediated activation. Porcine blood with added tPA showed significantly slower lysis compared to human blood. Additionally, purified porcine plasminogen was activated slowly by tPA in the presence of both human and porcine fibrin, with prolonged activation times in pigs compared to humans. Our study is comprehensive in not only determining the species specificity and cross-reactivity toward eventual plasmin generation but also characterizing the kinetics of plasminogen activation in a species-specific manner. Our study demonstrates that despite all the similarities between porcine and humans, particularly within the confines of the hemostatic system, there still exists a species-dependent difference in the overall efficacy in plasmin generation. Most notably, human fibrin provides a 10- to 25-fold greater plasmin generation compared with porcine fibrin as the cofactor without demonstrating any species-specific dependence of other fibrinolytic factors (i.e., similar catalytic efficiency). Alternatively, on porcine fibrin as the cofactor, there is a quantifiable difference between human and porcine plasminogen/tPA combination. Overall, our data suggest that the use of human tPA in pig system would result in at least a 5-fold reduction in overall catalytic efficiency in plasmin generation.

Differences were not limited to plasmin generation, but also in plasmin activity toward fibrin degradation. Porcine plasmin was consistently superior to human plasmin in fibrin clot digestion when assessed by turbidity. SDS-PAGE also demonstrated that there are subtle but clear differences in FDP fragment sizes generated between porcine and human fibrin(ogen) breakdown and relative accumulation of various FDP concentrations between human and porcine plasmin toward either fibrin(ogen). Porcine plasmin, relative to human plasmin, had a 2.7-fold and a 6.7-fold higher k _cat_ toward human and porcine fibrin, respectively. However, these differences were accompanied by similar increase in their K _m_ where porcine plasmin had a 2.8-fold and a 3.8-fold increase toward human and porcine fibrin, respectively. Although the overall catalytic efficiency (k _cat_ /K _m_ ) should be similar, it is likely that in most of our reaction conditions, and particularly at near physiologic concentrations of fibrinogen (approximately 9 µM), K _m_ is no longer as important and that the overall reaction would largely be determined by the differences in k _cat_ . However, higher activity of fibrin degradation by porcine plasmin would still not be sufficient to overcome the >5-fold lower catalytic efficiency in plasmin generation by human tPA, particularly in the presence of α _2_ AP to rapidly inhibit any plasmin that is generated *in situ* and in the presence of PAI-1 to inhibit tPA in plasma and whole blood systems, which explains the previous observations. 48

Comparison of primary structure of plasminogen, tPA, and fibrinogen between human and porcine sources also provided insights into the observed differences. For both plasminogen 11 12 13 14 and tPA, 40 41 the greatest differences were localized within their Kringle domains with reported key fibrin-binding properties. In plasminogen K1, of the key residues identified that are important for lysine binding, 42 43 49 50 there were two mutations identified—R32I and Y71F. In plasminogen K4, of the key residues identified for lysine binding, 51 52 only F64Y mutation from human to porcine was identified, with high conservation otherwise. Taken together, these changes could certainly impact functional outcome on its fibrin-dependent activation. Sequence analysis of tPA K2 domain revealed that although most of the key residues responsible for fibrin binding did not appear altered, 43 three consecutive residues in the area housing one of the key arginine residues were altered—NRR (76 to 78) to DHK. Fibrinogen also showed differences localized within its αC region, which has established binding sites for plasmin(ogen) and tPA, located largely in the αC region (392 to 610). 47 53 Despite poor alignment and sequence homology ( Fig. 7 ), majority of the key lysine and arginine residues in this region remained conserved ( Supplementary Fig. S3 ), suggesting that either source of fibrin(ogen) is able to promote plasminogen and tPA binding. Taken together, these data hint at the differences observed between human and porcine tPA for inducing fibrinolysis in porcine samples, and that the differences are highly localized and seemingly deliberate toward formation of the plasminogen/tPA/fibrin complex mediated through the lysine-dependent binding properties of plasminogen and tPA. Although it is unlikely that changes to one or two key residues within a functional domain is likely to have a large effect alone, cumulative changes across all three components may have a cumulative and/or multiplicative effect, seen in other complex formations. 54 Furthermore, since human fibrin appears to be superior to porcine fibrin as a cofactor, it is possible that the longer αC region of human fibrin offers higher affinity of plasminogen/tPA binding and/or an additional plasminogen/tPA binding site. Neither of these possibilities nor possible differences in the kinetics of direct inhibition of tPA by PAI-1 were explored further in our current study.

Our study provides a biochemical rationale for the observed differences between human and porcine fibrinolytic systems, particularly toward plasmin generation and consequent fibrin degradation. Despite overarching similarities in the fibrinolytic factors between the two species, the specific and localized differences within various domains lead to notable functional consequences. Understanding these differences are important particularly when animals are being used to model human diseases and their potential therapeutic developments.

## Notes

**What is Known About this Topic?**

- Porcine plasma clots are difficult to lyse using human tissue-type plasminogen activator (tPA).

**What Does this Paper Add?**

- Why porcine fibrinolytic system and human tPA have poor compatibility is uncertain.
- We show clear compatibility and their resulting kinetics of plasmin generation based on species of plasminogen, fibrin(ogen), and tPA.
- Largest variation between porcine and human proteins lies in fibrin-binding domains.
