# Supplementary material for: Differences and Compatibility between Human and Porcine Fibrinolytic Components toward Plasmin Generation and Fibrin Degradation
Source: TH Open. 2026 Jan 9;10:a27777484. doi: 10.1055/a-2777-7484 (PMC12817190; doi:10.1055/a-2777-7484)
Supplement: Supplementary file 1 — Supplementary Material [file 10-1055-a-2777-7484_27828756.pdf]

Thieme

Supplementary Figures and Tables

Differences and Compatibility between Human and Porcine Fibrinolytic Components toward Plasmin Generation and Fibrin Degradation (Kim et al.)

Table S1. Sequence alignment and analyses of human and porcine plasminogen by domain. NTP – N-terminal peptide; K – Kringle domains (1-5); PD – protease domain.

Domain	Length (Residues)	Identity	Similarity	Gap
NTP	79 (1 to 79)	56/79 (70.9%)	68/79 (86.1%)	0
K1	79 (84 to 162)	60/79 (75.9%)	67/79 (84.8%)	0
K2	79 (165 to 243)	69/79 (87.3%)	74/79 (93.7%)	0
K3	78 (256 to 333)	64/78 (82.1%)	72/78 (92.3%)	0
K4	78 (358 to 435)	66/78 (84.6%)	72/78 (92.3%)	0
K5	80 (462 to 541)	72/80 (90.0%)	75/80 (93.8%)	0
PD	228 (562 to 789)	190/228 (83.3%)	207/228 (90.8%)	0

Table S2. Sequence alignment and analyses of human and porcine tPA by domain. EGF – epidermal growth factor-like; K – Kringle domains (1-2); PD – protease domain.

Domain	Length (Residues)	Identity	Similarity	Gap
Finger	43 (17 to 59)	36/43 (83.7%)	39/43 (90.7%)	0
EGF	39 (60 to 98)	33/39 (84.6%)	35/39 (89.7%)	0
K1	82 (105 to 186)	67/82 (81.7%)	75/82 (91.5%)	0
K2	82 (193 to 274)	60/82 (73.2%)	69/82 (84.1%)	0
PD	251 (289 to 539)	219/251 (87.3%)	233/251 (92.8%)	0

Table S3. Sequence alignment and analyses of human and porcine fibrinogen alpha chain by domain.
FPA – fibrinopeptide A; αCC – αC connector; αCD – αC domain; CT – C terminus.

Domain	Length (Residues)	Identity	Similarity	Gaps
FPA	16 (1 to 16)	11/17 (64.7%)	14/17 (82.4%)	1/17 (5.9%)
Coiled	204 (17 to 220)	164/204 (80.4%)	190/204 (93.1%)	0
αCC	209 (221 to 391)	94/209 (45.0%)	109/209 (52.2%)	38/209 (18.2%)
αCD	231 (392 to 610)	118/231 (51.1%)	139/231 (60.2%)	32/231 (13.9%)
CT	237 (611 to 847)	215/237 (90.7%)	222/237 (93.7%)	0

Table S4. Sequence alignment and analyses of human and porcine fibrinogen beta chain by domain.
FPB – fibrinopeptide B; CT – C terminus.

Domain	Length (Residues)	Identity	Similarity	Gaps
FPB	14 (1 to 14)	6/21 (28.6%)	10/21 (47.6%)	7/21 (33.3%)
Coiled	66 (127 to 192)	59/66 (89.4%)	63/66 (95.5%)	0
CT	257 (202 to 458)	229/257 (89.1%)	245/257 (95.3%)	0

Table S5. Sequence alignment and analyses of human and porcine fibrinogen gamma chain by domain.
CT – C terminus.

Domain	Length (Residues)	Identity	Similarity	Gaps
Coiled	145 (3 to 147)	112/145 (77.2%)	132/145 (91.0%)	0
CT	261 (151 to 411)	229/261 (87.7%)	250/261 (95.8%)	0

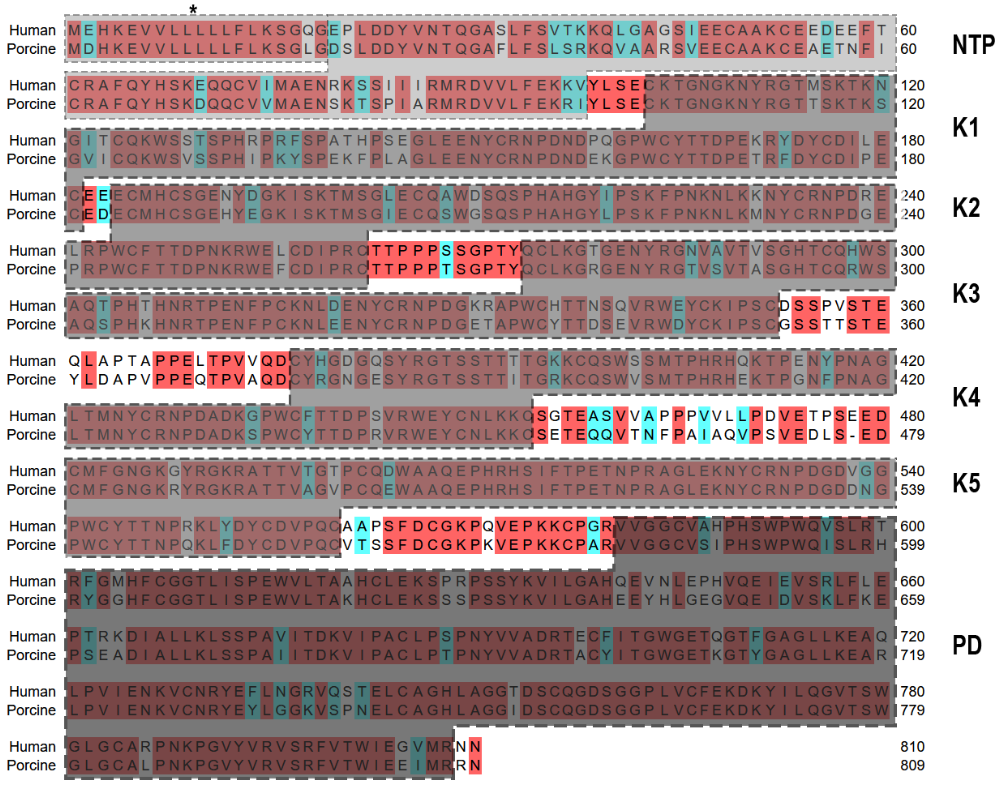

Figure S1. Comparison of the amino acid sequence of human (UniProtKB:P00747) and porcine plasminogen (UniProtKB:P06867). Comparison of the sequence shows 80% identity (red) and 88% similarity (red + green), with one residue omitted out of 810. The signal peptide (*), N-terminal peptide (NTP), five Kringle (K) domains, and the protease domain (PD) are highlighted.

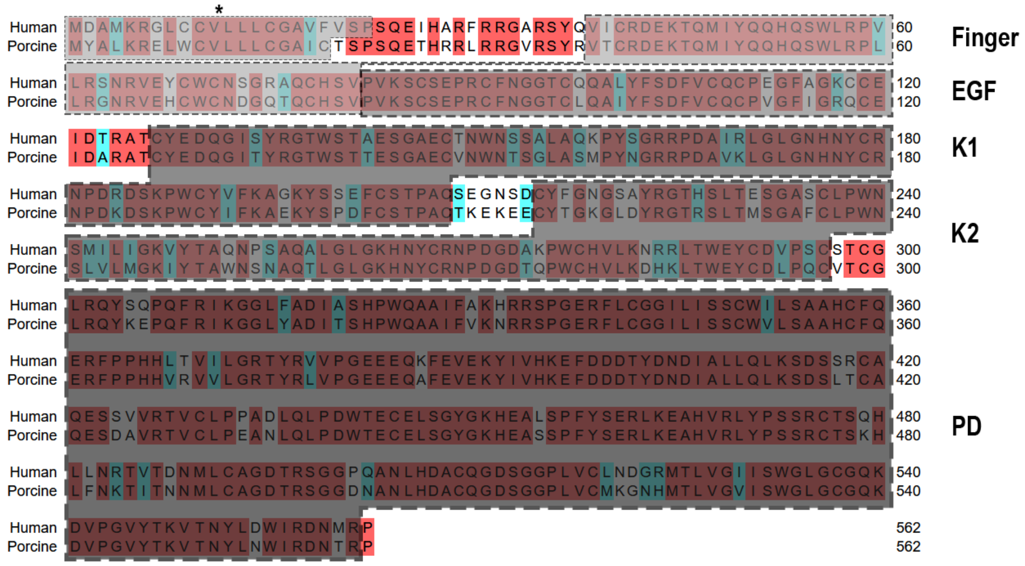

Figure S2. Comparison of the amino acid sequence of human (UniProtKB:P00750) and porcine tPA (UniProtKB:Q8SQ23). Comparison of the sequence shows 82% identity (red) and 89%

similarity (red + green), with 0 gap residues. * - signal peptide; Finger – Finger domain; EGF – epidermal growth factor-like domain; K – Kringle domains; PD – protease domain.

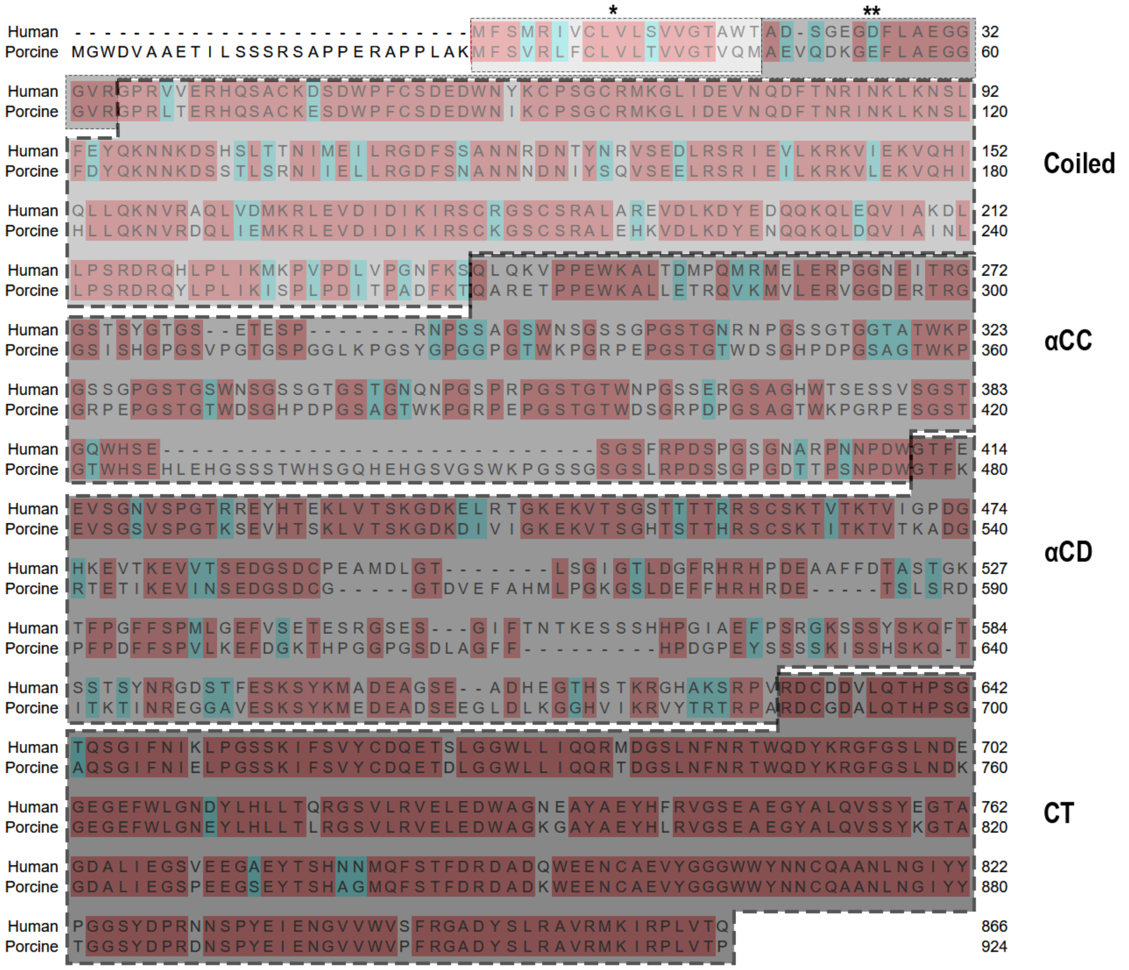

Figure S3. Comparison of the amino acid sequence of human (NP_000499.1) and porcine fibrinogen alpha-chain (XP_020957142.1). Sequence comparison resulted in a 65% identity (red) and 73% similarity (red + green), with 98 gap residues (10.4%). * - signal peptide; ** - fibrinopeptide A (FPA); αCC – αC connector; αCD – αC domain; CT – C terminal.

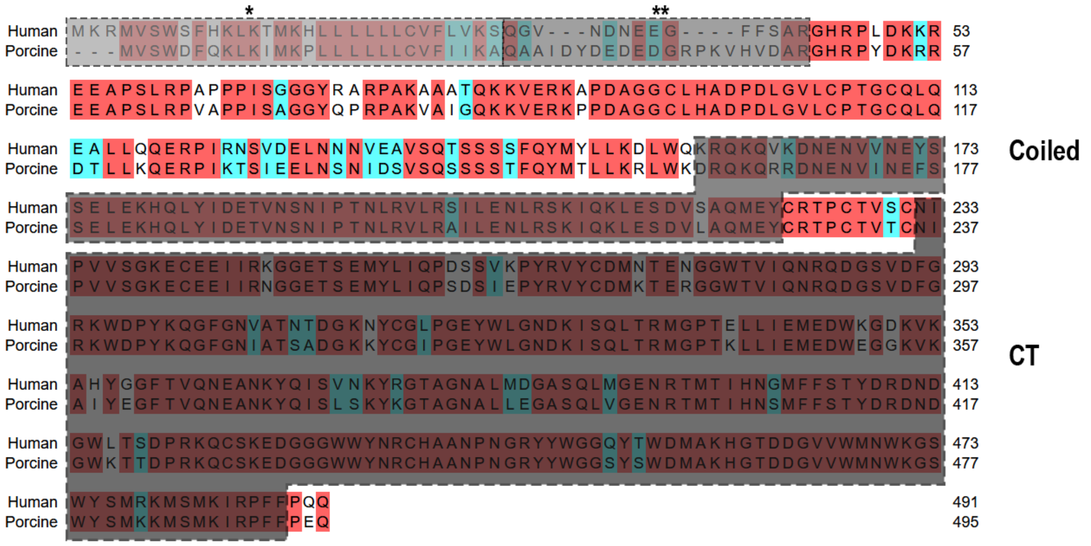

Figure S4. Comparison of the amino acid sequence of human (NP_005132.2) and porcine fibrinogen beta-chain (NP_001231042.1). Sequence comparison resulted in a 82% identity (red) and 90% similarity (red + green), with 10 gap residues (2%). * - signal peptide; ** - fibrinopeptide B (FPB), CT – C terminal.

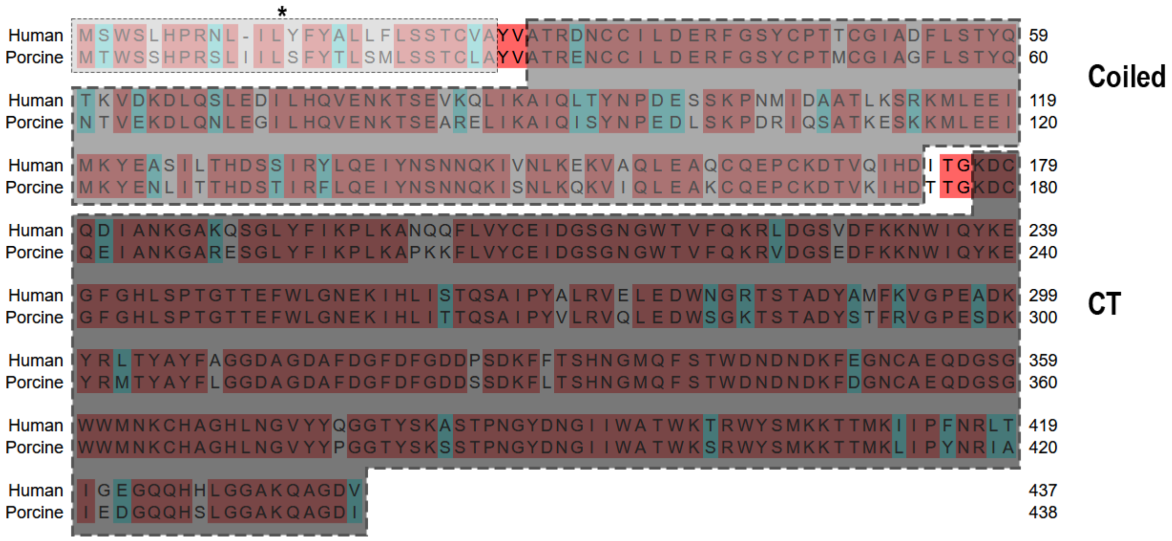

Figure S5. Comparison of the amino acid sequence of human (NP_000500.2) and porcine fibrinogen gamma-chain (NP_001231453.1). Sequence comparison resulted in a 83% identity (red) and 93% similarity (red + green), with 1 gap residue (0.2%). * - signal peptide; CT – C terminal.

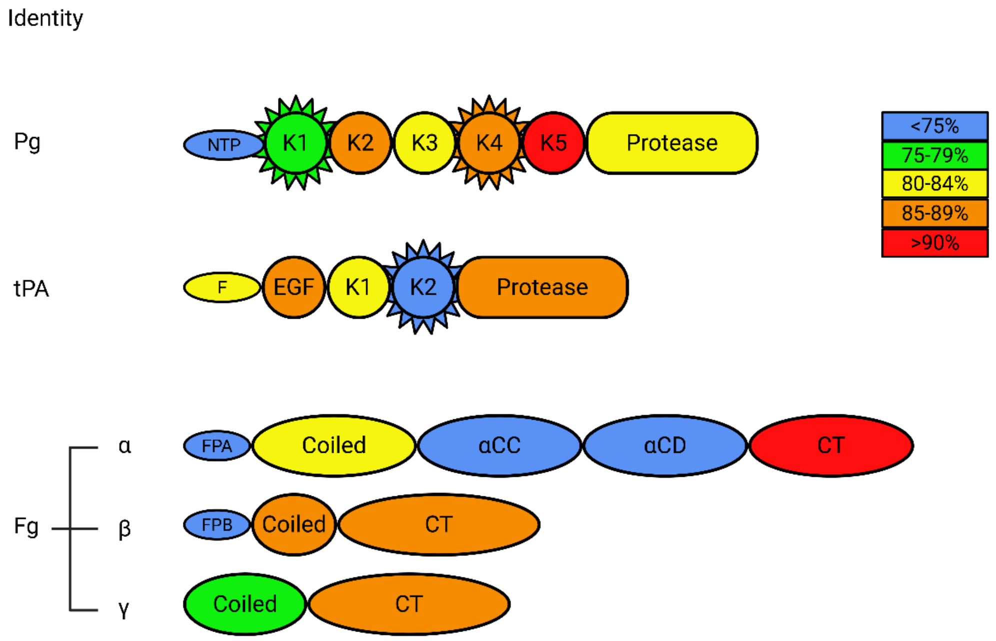

Figure S6. Identity of amino acid sequences when compared between porcine and human plasminogen, tPA, and fibrinogen chains by domain. The heat map illustrates domains that display highest (red) to lowest (blue) identical residues. The spikes highlight the domains that bind fibrinogen in a lysine-dependent manner. Created in BioRender. Kim, P. (2025) https://BioRender.com/ucp8l0e
